# Unlocking glioblastoma: breakthroughs in molecular mechanisms and next-generation therapies

**DOI:** 10.1007/s12032-025-02830-1

**Published:** 2025-06-21

**Authors:** Fariah Rizwani, Pallavi Patil, Khush Jain

**Affiliations:** 1 Department of Pharmacology, Bharati Vidyapeeth College of Pharmacy, University of Mumbai, Mumbai, India; 2 Pharmacology and Therapeutics, School of Medicine, University of Galway, Galway, Ireland

**Keywords:** Glioblastoma, Systematic review, Immunotherapy, Tumor microenvironment, Therapeutic resistance, Targeted therapy

## Abstract

Glioblastoma (GB) remains the most aggressive primary brain tumor in adults, characterized by rapid progression, recurrence, and resistance to conventional therapies. Despite advancements in surgical resection, radiation, and chemotherapy, long-term survival rates remain low. This review comprehensively explores GB’s molecular classification, pathological mechanisms, epidemiology, and emerging therapeutic strategies. Key genetic mutations in TP53, MAPK/ERK, PI3K/AKT/mTOR, and many more signaling pathways, such as Notch, Wnt, Hedgehog, TGF-β, and NF-κB drive tumor progression, therapy resistance, and immune evasion. Diagnostic advances, including multi-modal imaging and molecular profiling, have improved early detection and precision therapy selection. Conventional treatments such as temozolomide and radiation therapy provide modest benefits, but novel approaches offer promising alternatives. Immunotherapy, targeting checkpoint inhibitors and tumor vaccines, has emerged as a potential avenue for enhancing tumor control. Nanotechnology-based drug delivery, particularly liposomal formulations and CRISPR-Cas9 gene editing improves blood–brain barrier penetration and reduces systemic toxicity. Targeted inhibitor-based therapies, including angiogenesis inhibitors, help limit tumor vascularization. Furthermore, a systematic review of 16 clinical trials highlights the emerging trends in combinatorial strategies, their adverse events, and outcomes, which remain pivotal for optimizing GB management. This review synthesizes current research while emphasizing future directions that could revolutionize GB therapeutic approaches and improve patient survival.

## Introduction

Glioblastoma (GB) continues to be the most prevalent and aggressive primary brain tumor in adults, resulting in approximately 60% of all brain tumors [1]. Despite advancements in surgical, radiation, and chemotherapeutic strategies, GB is still considered highly lethal, with a median survival rate of only 5–10% over 5 years [2]. Its rapid progression, recurrence, and therapeutic resistance make it one of the most challenging cancers to manage clinically. GB arises from a complex biology of genetic mutations, environmental exposures, and metabolic alterations. Established risk factors include high-dose ionizing radiation, obesity, chronic inflammation, and a family history of gliomas [3]. While cytomegalovirus (CMV) has been suggested as a potential contributing factor, its precise role in GB remains controversial due to conflicting studies [4]. Certain hereditary syndromes, such as Li-Fraumeni Syndrome (TP53 mutations) and Neurofibromatosis Type 1 (NF1 mutations), significantly increase glioma risk by disrupting tumor suppressor pathways [5]. Additionally, IDH1/2 mutations, commonly observed in secondary GB, are associated with a better prognosis compared to IDH-wildtype GB, which is more aggressive. TERT promoter mutations, linked to telomerase activation, promote tumor immortality and therapy resistance, further complicating disease management [6]. Large-scale genomic studies, including The Cancer Genome Atlas (TCGA), evaluated over 600 genes from approximately 200 human tumor samples and have identified key signaling disruptions in TP53, MAPK/ERK, PI3K/Akt/mTOR, and EGFR pathways, which collectively drive tumor proliferation, invasion, and therapeutic resistance. Chromosomal instability, such as EGFR amplification and combined chromosome 7 gain/chromosome 10 loss, also plays a significant role in tumor progression and treatment response [7, 8]. GB’s highly invasive nature makes complete surgical resection nearly impossible, as tumor cells infiltrate surrounding brain tissue, leading to inevitable recurrence [9]. Additionally, the tumor exhibits intrinsic resistance to chemotherapy and radiation, limiting long-term treatment success. Standard-of-care therapies, including temozolomide and bevacizumab, provide only modest survival benefits, necessitating urgent exploration of novel therapeutic interventions [10]. Emerging strategies, including immunotherapy, targeted molecular therapies, and combinatorial approaches, aim to enhance survival outcomes by counteracting therapy resistance mechanisms [11]. Recent advances in biomarker discovery offer promising avenues for early detection, precision treatment planning, and prognostic stratification, which could revolutionize GB management in clinical practice [12]. This review aims to provide a comprehensive synthesis of GB molecular biology, signaling pathways, and therapeutic advancements, with a particular emphasis on emerging genetically targeted interventions and innovative treatment paradigms. Through a systematic evaluation of clinical trials, we will highlight promising therapies with potential real-world applicability, while addressing future perspectives in GB management and therapeutic optimization.

## Materials and methods

### Search strategy

A detailed literature review was performed across PubMed, Scopus, and Web of Science databases to identify studies examining glioblastoma molecular classification, epidemiology, etiology, molecular mechanisms, signaling pathways, and therapeutic interventions. Additionally, a systematic review of emerging therapies was done using the ClinicalTrials.gov database, focusing on ongoing clinical trials [13–16]. The search focused on publications between 2011 and 2025, prioritizing peer-reviewed articles and randomized clinical trials for inclusion.

### Study selection process

**Fig. 1:**
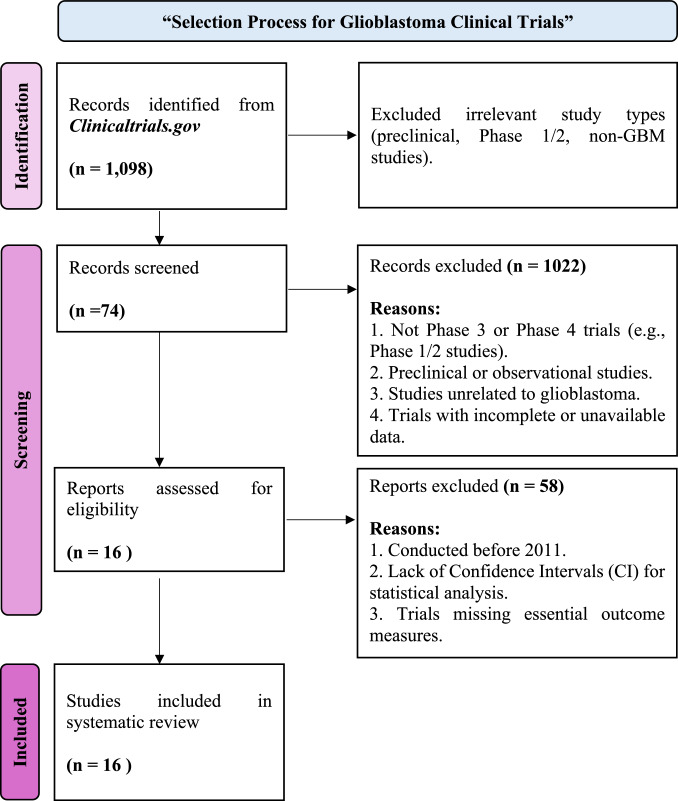
Flow Diagram for Study Selection The flow diagram represents the systematic review of studies, detailing the number of records identified, screened, excluded, and included in the final analysis

The selection process for the systematic review followed a stepwise approach, including identification, screening, eligibility assessment, and final inclusion of studies. The workflow, outlining article selection and refinement, is illustrated in Fig. 1.

### Molecular classification of GB

**Fig. 2:**
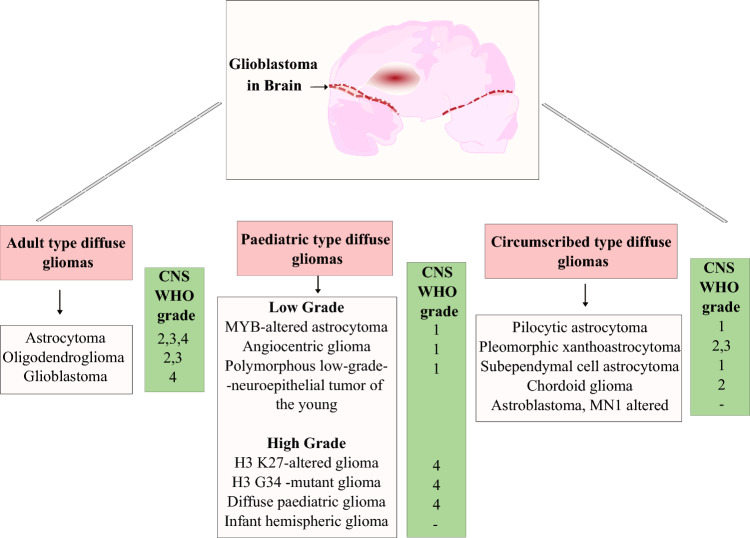
Classification of the glioma within each tumor type, based on molecular and genetic characteristics It categorizes gliomas into three primary groups: adult-type diffuse gliomas, pediatric-type diffuse gliomas, and circumscribed gliomas. GB falls under Grade IV adult-type diffuse gliomas, demonstrating high aggressiveness and recurrence rates [18]

GB is classified within the central nervous system (CNS) tumor grading system, categorized based on the amount of invasiveness into the brain and its surrounding tissues—diffuse gliomas and circumscribed gliomas [17]. The 2021 World Health Organization (WHO) Classification of CNS Tumors categorizes gliomas into adult-type diffuse gliomas, pediatric-type diffuse gliomas, and circumscribed gliomas, with GB classified as a Grade IV malignancy due to its high recurrence rate and aggressive nature [18]. Adult-type diffuse gliomas, including astrocytoma, oligodendroglioma, and glioblastoma, exhibit extensive infiltration into the brain parenchyma, making surgical resection challenging. Pediatric-type diffuse gliomas, such as H3 K27-altered gliomas, have distinct molecular features and treatment responses compared to adult gliomas. Circumscribed gliomas, including pilocytic astrocytomas and pleomorphic xanthoastrocytomas, are less invasive and often have better prognostic outcomes. Histologically, diffuse gliomas could be oligodendroglial or astrocytic, with astrocytoma being the most prevalent subtype, responsible for 50% of all malignant brain tumors [19]. The complete classification structure of gliomas is illustrated in Fig. 2.

### Morphological variants of GB

GB presents a diverse range of morphological traits, contributing to its heterogeneity, invasiveness, and therapeutic resistance. Characteristic histopathological features include microvascular proliferation, cellular pleomorphism, mitotic activity, and nuclear atypia, which collectively enhance tumor progression and adaptability to treatment pressures. The presence of these features complicates surgical resection and often correlates with aggressive disease behavior [20]. Additionally, GB presents diverse histological variants that further complicate treatment strategies. These rare subtypes exhibit unique molecular characteristics influencing tumor growth and therapy response. For instance, gliosarcomas possess both astrocytic composition alongside mesenchymal traits, mimicking sarcoma-like behavior. This distinct subtype exhibits aggressive invasiveness and heightened resistance to conventional therapies, demanding specialized treatment approaches that target both epithelial and mesenchymal tumor components [21]. Another distinct variant, giant-cell glioblastomas, is characterized by large, multinucleated tumor cells, which contribute to abnormal cellular architecture and proliferation patterns. This subtype represents an uncommon yet notable deviation from conventional GB pathology, requiring specific diagnostic markers to differentiate its biological behavior from typical glioblastomas [22]. Additionally, small-cell glioblastomas are frequently associated with epidermal growth factor receptor (EGFR) amplification, a well-documented driver of tumor aggressiveness and therapeutic resistance. EGFR alterations accelerate uncontrolled cellular replication, fueling rapid progression and diminishing the effectiveness of EGFR-targeted therapies [7]. The presence of small-cell components in GB may warrant alternative molecular-targeted approaches to improve treatment outcomes [23]. The existence of these distinct histological subtypes underscores the complexity of GB classification and reinforces the necessity for precise molecular profiling in therapeutic decision-making. Recognizing variant-specific tumor behavior allows clinicians and researchers to refine personalized treatment strategies, optimizing patient management and improving survival outcomes.

### Etiology of GB

GB has a multifaceted etiology, influenced by various factors such as environmental, genetic, and lifestyle factors. Radiation therapy elevates the risk of progressing GB, having exposure to the head and neck, causes DNA damage, leading to further mutations and alterations in genomic cells, eventually causing instability and tumorigenesis [24]. Studies have shown that young adults previously exposed to radiation therapy for other conditions exhibit a higher likelihood of GB development later in life. Occupational exposures to hazardous chemicals, synthetic rubber, and vinyl chloride production have an increased risk of GB due to the neurotoxic effects of these toxins. These induce cellular impairment, apoptosis, and even DNA mutations [25]. Additionally, prolonged exposure to polluted air and carcinogens like benzene has resulted in neuro-oncological diseases [25]. Genetically, individuals with Li-Fraumeni syndrome, caused by the genetic alterations in the TP53 gene, considerably elevated risk of GB, as this gene plays a pivotal role in regulating cell functioning and its apoptosis. Dysfunctional TP53 leads to unrestricted cellular proliferation, facilitating tumor progression [24]. Similarly, Neurofibromatosis (NF1 and NF2) is caused by genetic alterations that produce tumor suppressor protein, associated with GB development, further highlighting the importance of genetic predisposition [26]. Tuberous Sclerosis Complex (TSC) is another genetic disorder caused by mutations in the TSC1 or TSC2 genes, expediting the growth of non-cancerous tumors. TSC is primarily linked to benign tumors, including glioblastoma, due to the alterations in signaling pathways. These mutations affect cell maintenance, cell cycle regulation, and cellular processes of metabolism. Such genomic variations are often associated with gliomas and tumorigenesis [27]. Lifestyle factors, particularly cigarette smoking, have been associated with various types of cancer, and recent studies have suggested that it may increase in risk of developing gliomas. The toxins and carcinogens emitted by tobacco smoke induce oxidative stress and inflammation, which may lead to tumor proliferation and a decline in survival rate in susceptible individuals [28]

### Epidemiology of GB

GB affects populations worldwide, with an estimated global incidence rate of 3.33 cases per 100,000 individuals [29]. However, significant differences in the demographic and geographic patterns could regulate its incidence and prognosis. GB affects populations worldwide, with an estimated global incidence rate of 3.33 cases per 100,000 individuals. However, significant geographical, demographic, and ethnic disparities influence disease prevalence and prognosis [30, 31]. The highest incidence is observed among individuals aged 55–60 years, aligning with peak susceptibility due to cumulative genetic alterations and environmental exposures. Additionally, males demonstrate higher GB prevalence than females, suggesting that biological sex-linked factors may contribute to tumor initiation and progression. Racial/ethnic differences also play a critical role in the incidence and prognosis of GB [30, 31]. Caucasians experience higher GB incidence rates compared to African Americans, Asians, and Hispanics, though the underlying genetic and environmental mechanisms remain under investigation [32]. Moreover, differences in healthcare accessibility, socioeconomic status, and early detection rates contribute to variations in survival outcomes across populations [33]. GB is more prevalent in Western countries due to better healthcare facilities and early detection compared to other countries globally [30, 31]. Environmental risk factors, such as exposure to radiation (head and neck), can also increase the chances of GB, as the carcinogens from the environment play a vital role in tumorigenesis. Despite ongoing therapeutic advancements, GB prognosis remains poor, with an overall 5–10% survival rate over five years, posing a significant challenge for researchers and clinicians in developing effective long-term treatment strategies [34].

### Pathophysiology of GB

GB is an aggressive and genetically heterogeneous glioma, characterized by its immune evasion and rapid proliferation. Its pathophysiology encompasses a series of molecular, genetic, and metabolic alterations that contribute to the tumor progression and resistance to conventional therapeutic strategies [35]. GB is a complex interaction of genetic mutations, metabolic disruptions, signaling pathways, and tumor microenvironment (TME). These alterations advance the malignant phenotype of GB, leading to complicated therapeutic strategies and rapid deterioration in patients’ health [36]. Epidermal growth factor receptor (EGFR) mutations are one of the hallmark alterations of GB, mainly in the IDH-wildtype subtype, or the mutations of the EGFRvIII variant, leading to activation of the receptor, causing uncontrolled tumor growth and metabolic dysregulation even in the absence of a ligand. Overexpression of EGFR activates PI3K/AKT/mTOR signaling, promoting tumor invasiveness and resistance to targeted therapies. Furthermore, the predominant activation of pathways induces resistance to therapies [37]. The TP53 tumor suppressor gene, a critical tumor suppressor, is responsible for cell cycle regulation and apoptosis due to oxidative stress or DNA damage. Furthermore, GB causes mutations in TP53, inducing uncontrolled cellular proliferation and promoting tumor growth [38]. This instability further enables genomic changes, facilitating tumor evolution. Additionally, alterations in retinoblastoma protein 1 (RB-1), which regulates the cell cycle (particularly G1-S transition), are also altered, contributing to GB’s aggressive behavior. These dysfunctions in both TP53 and RB-1 create a permissive environment for tumor progression and therapy resistance [35, 36]. Distinctively, isocitrate dehydrogenase (IDH) mutations are found in secondary GB, inducing the accumulation of oncometabolite 2-hydroxyglutarate (2-HG). It inhibits α-ketoglutarate-dependent dioxygenases, leading to epigenetic dysregulation and altered cellular metabolism [39]. Additionally, the TERT Promoter is responsible for telomere maintenance, but mutations in TERT cells drive telomerase enzyme overactivation, which eventually affects GB cells. These mutations cause tumor cell immortality and continued proliferation. These alterations collectively contribute to GB’s resistance to apoptosis and its ability to evade therapy [39]. GB is also dependent on angiogenesis, primarily through the upregulation of the Vascular Epithelial Growth Factor (VEGF). It facilitates cell proliferation, due to the formation of leaky and dysfunctional blood vessels, providing nutrients and oxygen, and supporting the tumor’s survival. However, these abnormalities impair effective drug delivery, limiting the efficacy of targeted treatments [40]. GB impairs the immune system through the activation of tumor-associated macrophages (TAMs), which cause inflammation via inflammatory cytokines (TGF-β and IL-10). These inflammatory cytokines promote angiogenesis and tumor progression by inhibiting T cells and natural killer (NK) cells [41]. GB cells infiltrate surrounding brain tissues and are promoted by matrix metalloproteinases (MMPs), which deteriorate the extracellular matrix layer for easier penetration of tumor cells in adjacent tissues [42]. Alternatively, there’s perivascular invasion, providing a supportive microenvironment for tumor cells, helping them proliferate in the central nervous system, and impacting the blood–brain barrier (BBB). This local invasion contributes significantly to the challenges of treating GB, as tumor cells can infiltrate critical brain regions, complicating surgical and therapeutic interventions [43].

### Molecular and genetic profiling of GB

Molecular and genetic profiling has become an important technique in the diagnosis, prognosis, and treatment of GB. Due to its highly heterogeneous nature, deeper molecular insights are essential for patient stratification and developing personalized treatment strategies [44]. The genomic studies, such as The Cancer Genome Atlas (TCGA), have identified distinct molecular subtypes, Proneural, Mesenchymal, and Classical GBM, each exhibiting unique genetic landscapes that shape tumor progression and response to treatment [45]. Profiling techniques, including next-generation sequencing (NGS), DNA methylation analysis, and single-cell transcriptomics, have refined GBM classification by identifying critical genetic alterations associated with tumor aggressiveness and therapy resistance. Particularly, the genetic mutations observed, such as TP53 mutations, have led to apoptotic dysregulation, compromising genomic stability and enabling unchecked tumor proliferation despite chemotherapy and radiotherapy-induced stress [46]. Research is investigating MDM2/MDM4 inhibitors to restore TP53 function in mutant GBM. EGFR amplifications, particularly the EGFRvIII variant, are frequent in GBM, driving constitutive activation of PI3K/AKT/mTOR and MAPK/ERK pathways, which sustain tumorigenesis even in the absence of ligands [47]. IDH mutations, primarily observed in secondary GBM, result in oncometabolite 2-hydroxyglutarate (2-HG) accumulation, altering histone methylation and cellular differentiation [47, 48]. IDH-mutant tumors generally exhibit better responses to alkylating agents and radiation, with inhibitors like ivosidenib currently being explored in clinical trials [49]. TERT promoter mutations drive telomerase overactivation, ensuring tumor longevity by evading cellular aging processes, contributing to resistance mechanisms, and recurrence. Telomerase inhibitors and immune-based therapies targeting TERT-expressing cells are under investigation as potential GBM treatments [50]. Additionally, GBM exhibits significant chromosomal instability, with characteristic gain of chromosome 7 and loss of chromosome 10, contributing to genomic instability and therapy resistance. Single-cell sequencing approaches are increasingly being used to define subclonal tumor populations, aiding the development of targeted interventions based on patient-specific genetic profiles [51]. One of the major clinically relevant biomarkers is the O6-methylguanine-DNA methyltransferase (MGMT) gene promoter. MGMT is a DNA repair enzyme that reverses the DNA damage. The epigenetic silencing of MGMT via promoter methylation disrupts its capability to repair DNA, which in turn enhances the inducer’s sensitivity [e.g., Temozolomide(TMZ)] [52]. In MGMT-methylated GB cell lines (e.g., U87MG, LN229), phenothiazines and TLK1 inhibitors have shown synergistic effects with TMZ by diminishing the DNA repair mechanisms, reducing tumor growth, and its invasion. Whereas MGMT-unmethylated cells (e.g., LN18) retain DNA repair mechanisms, and can respond to TLK1 inhibitors alone, to highlight its potential in TMZ-resistant GB [52]. Despite these recent advances, GB remains challenging due to its tumor heterogeneity. It displays both intratumoral and intertumoral variability, with co-existing subclones harboring distinct mutations and epigenetic landscapes [53]. Spatial differences within a single tumor complicate its treatment strategies, as resistant populations could emerge and expand following standardized therapy. The tumor microenvironment (TME) is another layer of complexity for therapies. Glioma stem-like cells (GSCs) and tumor-associated macrophages (TAMs) contribute to treatment resistance by promoting MGMT expression, ABC transporter activation, and enhancing immune evasion [54]. These microenvironmental interactions sustain a resistant, invasive phenotype and undermine responses to both cytotoxic and immunomodulatory therapies [55]. MGMT promoter methylation status guides TMZ use, but such resistance makes it challenging, which often arises from ABC transporters (e.g., ABC1 and ABCC1) or GSC-associated repair mechanisms [56]. Additionally, poor T cell infiltration via the BBB hinders the efficiency of immune checkpoint blockade [56]. Integrating genomics, multi-omics, proteomics, and transcriptomics with clinical data may support personalized treatment [57]. Importantly, tumor heterogeneity challenges conventional clinical trial design. Standardized protocols often fail to account for patient-to-patient variability, potentially obscuring treatment efficacy. Adaptive and basket trial frameworks, incorporating molecular profiling into patient selection, are increasingly being adopted to better stratify patients and evaluate targeted therapies [58].

### Cell signaling pathways of GB

**Fig. 3:**
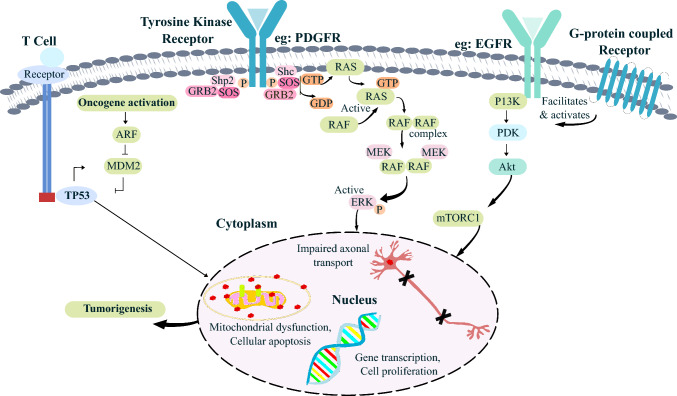
Schematic representation of dysregulation of key signaling pathways in GB This diagram illustrates the disruption of the T cell receptor, receptor tyrosine kinase (RTK)/MAPK/ERK, and P13K/Akt/mTOR signaling cascades in GB. The unusual activation of these pathways distorts cellular processes and impairs axonal transport, gene transcription, cell proliferation, mitochondrial function, and apoptosis of cells. The cumulative effect of these molecular alterations leads to tumorigenesis in GB [73, 74]

The malignant progression and therapeutic resistance of GB arise from the disruptions in key intracellular signaling pathways, primarily TP53, MAPK/ERK, and PI3K/AKT/mTOR. These pathways govern tumor survival, proliferation, immune modulation, and metabolic adaptation, contributing to GB’s aggressive nature and resilience to conventional treatments [59]. The TP53 pathway, commonly referred to as the genomic guardian, is responsible for DNA repair, apoptosis, and cell cycle control. Mutations in TP53, along with CDKN2A/ARF deletions, disrupt tumor suppression mechanisms, allowing unchecked proliferation and therapy resistance [60, 61]. The MDM2/MDM4 axis plays a critical role in TP53 degradation, and MDM2 inhibitors are being studied as potential strategies to restore p53 pathway activity [62]. However, despite promising experimental approaches, TP53-targeted interventions face challenges due to tumor heterogeneity, compensatory survival mechanisms, and lack of sustained efficacy [61]. The MAPK/ERK pathway comprising RAS, RAF, MEK, and ERK kinases is frequently hyperactivated via receptor tyrosine kinases (RTKs), such as EGFR and PDGFR [59]. Persistent signaling within this cascade drives tumor proliferation, survival, and invasive potential while contributing to immune evasion through glioblastoma-associated macrophages (TAMs) and inflammatory cytokines (IL-6, IL-8, TGF-β). The pathway modifies the extracellular matrix (ECM) via matrix metalloproteinases (MMPs), facilitating tumor infiltration into surrounding brain tissue. Therapies targeting MEK and ERK inhibitors have shown limited efficacy due to redundant oncogenic networks, requiring combination strategies integrating RTK blockade, immune modulation, and ECM-targeting agents [63, 64]. The PI3K/AKT/mTOR pathway is closely linked with MAPK/ERK and controls tumor metabolism, survival, and therapy resistance. Loss of PTEN, a tumor suppressor, leads to aberrant PI3K activation, driving PIP3 accumulation and AKT phosphorylation, which in turn modulates apoptotic resistance and protein synthesis via mTORC1/mTORC2 signaling [65]. Dysregulation of this pathway enables GB cells to evade cell death, fuelling therapy resistance. While PI3K inhibitors have been explored, therapeutic success is hindered by feedback activation and compensatory metabolic shifts, reinforcing the necessity of multi-targeted treatment approaches. These signaling networks are deeply interconnected, reinforcing GB’s adaptive survival strategies. Their convergence at RTKs and downstream effectors enables rapid signaling adjustments, and counteracting single-agent therapies [66]. Understanding these complex molecular interactions is critical for designing effective therapeutic interventions, incorporating RTK inhibitors, immune modulation, metabolic rewiring, and ECM-targeted treatments. A schematic overview of these major dysregulated pathways is presented in Fig. 3. In addition to these major signaling pathways, several other signaling pathways contribute to the pathogenesis of GB. The Notch signaling cascade regulates cell fate decisions, and it gets activated in GSCs. This cascade is responsible for tumor maintenance, relapse, and therapeutic resistance [67]. Notch receptors undergo cleavage upon ligand binding (e.g., Delta or Jagged) by the γ-secretase enzyme. This releases the notch intracellular domain (NICD), which translocates to the nucleus and causes gene transcription. In GSCs, Notch activation enables self-renewal and chemoresistance. γ-secretase inhibitors have shown a significant effect in pre-clinical studies by impairing GSC survival, but this has limited their clinical utility due to gastrointestinal toxicity or acquired resistance [68]. The Wnt or β-catenin signaling pathway is another pivotal regulator of GB. It influences tumor invasion, proliferation, and stemness. Wnt ligands interact with LRP and Frizzled receptors, which inhibit the β-catenin destruction and allow it to translocate to the nucleus. Nuclear β-catenin then promotes malignancy via gene transcription. Therapeutic approaches targeting β-catenin, Wnt ligands, and their complexes are under investigation [69]. The Hedgehog signaling pathway is often abnormally activated in GB, supporting tumor growth, therapy resistance, and stemness. It is mediated by smoothened (SMO) and GLI transcription factors. SMO activation leads to nuclear localization of GLI (1/2/3) proteins, regulating gene expression, thus linked to tumor survival. Additionally, non-canonical Hh activation and alternative GLI-1 isoforms further complicate pathway inhibition. Although SMO and GLI factor inhibitors are still under development, SMO mutations and bypass activations make it challenging for clinical impact [70]. The TGF-β signaling initially behaves as a tumor suppressor, inducing apoptosis and cell cycle arrest, but in later stages, it promotes epithelial-mesenchymal transition (EMT) and angiogenesis. TGF-β supports the GSCs’ tumor microenvironment to suppress the anti-tumor immunity. Therapies are being investigated to selectively disrupt the tumor-producing effects [71]. Finally, the NF-κB pathway gets activated and it releases pro-inflammatory responses, promoting GB cell invasion, and therapy resistance through its anti-apoptotic and upregulating cytokine properties. Recent developments are being explored to sensitize these receptors, which could reverse immune suppression and improve patient outcomes [72].

#### Clinical presentation of GB

The clinical presentation of GB depends on multiple factors such as the tumor’s size, location, and the rate at which it is progressing. People start experiencing symptoms like headaches, migraine, and stress due to increased intracranial pressure, these deficits trigger vomiting and visual disturbances [75]. These neurological deficits are particularly attributed to the invasiveness of GB and the part of the brain it affects. Disruption of the frontal lobe affects the person’s personality, impulsivity, and cognitive impairments such as challenges to memorize, prolonged attention, and function [75]. These symptoms are often mistaken for psychiatric disorders or normal aging. Gliomas even affect the temporal lobe, which can lead to auditory hallucinations, language aphasia, and severe memory loss. Damage to the parietal lobe can result in sensory deficits, impacting patients’ ability to respond to stimuli on the lateral side of the body [76]. Cerebellar impairment affects coordination difficulties, gait disruption, including ataxia, and complications with balance [75, 77]. Seizures are also commonly found symptoms in GB, which accounts for 20–40% of the patients globally if the GB affects the brain areas for sensory and motor processing. The distress to the brain due to tumors enhances the risk of seizures and epileptic disorders. However, patients with migraine, headaches, and other brain tumors (e.g., meningioma, metastatic tumors), infections (e.g., encephalitis, abscess), vascular abnormalities (e.g., arteriovenous malformations), autoimmune or inflammatory conditions, and neurodegenerative diseases (e.g., Alzheimer’s disease, Parkinson’s disease) [75, 76] must be examined for GB at the earliest.

#### Diagnostic approaches for GB

Early and accurate diagnosis of GB is essential for guiding treatment strategies and improving patient outcomes. A multi-modal diagnostic approach is now considered standard practice, combining advanced imaging techniques such as Magnetic Resonance Imaging (MRI), Computed Tomography (CT), and Positron Emission Tomography (PET), as histopathological evaluation, and real-time intraoperative monitoring to enhance precision and evaluate tumor size, location, and progression [78]. MRI remains the gold standard for GB imaging, providing high-resolution, multiplanar images with superior soft tissue contrast. It also provides comprehensive structural and physiological insights through T2-weighted, Fluid-Attenuated Inversion Recovery (FLAIR), pre-gadolinium, and post-gadolinium T1-weighted imaging, as well as advanced sequences such as diffusion and perfusion-weighted imaging (DWI and PWI), diffusion tensor imaging (DTI), and MR spectroscopy [79]. To enhance diagnostic accuracy, early post-operative MRI is recommended no later than 72 h; this minimizes confusion and helps in assessing the residual tumor and monitoring response [80]. DWI helps locate regions of high cellularity and differentiate infarct-related changes from residual tumor, whereas PWI quantifies blood flow metrics, especially dynamic susceptibility contrast (DSC), which focuses on relative cerebra blood volume (rCBV), which can help in differentiating true progression from pseudo-progression [81]. CT is less sensitive than MRI but valuable in emergency settings or when MRI isn’t satisfactory. CT being less detailed, can be useful for detecting calcification or hemorrhage. PET imaging, particularly in combination with MRI or CT, provides metabolic insights using radiotracers like fluorodeoxyglucose (FDG) and O-(2-[18F]fluoroethyl)-L-tyrosine (FET), which help assess tumor aggressiveness, therapy response, and distinguish tumor progression from treatment-related changes [82]. Histopathological confirmations remain central to GB diagnosis. Tissue biopsy or resection samples are evaluated for classical features such as nuclear atypia, necrosis, and microvascular proliferation [83]. Immunohistochemistry (IHC) is another cost-effective and rapid technique to identify diagnostic and prognostic markers such as IDH1, p53, EGFR, and Ki-67 [83, 84]. Advances in molecular diagnostics also allow for better tumor classification and personalized therapeutics. Additionally, next-generation sequencing (NGS) is widely used to identify mutations in TP53, EGFR, PTEN, TERT, and others [85]. MGMT promoter methylation is detectable via methylation-specific PCR or its sequencing [86]. Liquid biopsy is another diagnostic technique that works especially through circulating tumor DNA (ctDNA) analysis. This technique has emerged as a promising non-invasive alternative to tissue biopsy, as it enables longitudinal monitoring, detection of minimal residual disease, and real-time tracking of treatment response and resistance [87]. The utility of ctDNA from blood in GB is limited to the BBB [88]. Cerebrospinal fluid offers superior sensitivity for detecting CNS tumor mutations [87]. Other research studies have also investigated urine, saliva, pleural effusions, and bile, but thorough validation is still essential [89]. Technological platforms such as NGS, methylation profiling, and digital droplet PCR are enhancing traditional accuracy, but the challenges persist in standardization and clinical implementation [90]. Intraoperative neurophysiological monitoring (IONM) is increasingly integrated in targeting glioma, specifically for those in the eloquent cortex. To preserve neurological function by mapping and protecting critical motor and sensory pathways employing techniques such as somatosensory-evoked potentials (SSEPs), transcranial motor-evoked potentials (TcMEPs), and direct cortical stimulation at the time of resection surgeries [91]. The combination of IONM, with DTI and MRI, enables precise resection, also minimizing post-operative deficits. IONM could preserve functional outcomes and help in improving quality of life, but it doesn’t directly impact overall survival [91]. Overall, a comprehensive diagnostic strategy could be beneficial for GB employing liquid biopsy and IONM, which enhances precision and personalized treatment to improve patient outcomes.

#### A systematic review of clinical trials for GB: Emerging therapies & their potential

GB remains one of the most treatment-resistant brain tumors, with limited long-term survival despite advances in standard care. In response to the challenges of tumor recurrence, therapy resistance, and patient heterogeneity, numerous clinical trials have explored a broad spectrum of therapeutic strategies. These include immunotherapy (e.g., nivolumab, ipilimumab, DSP-7888), targeted therapies (e.g., ABT-414, Marizomib, Imatinib), optimized radiotherapy protocols, and novel combinations of chemotherapy agents such as temozolomide, veliparib, and cediranib. Clinical trials continue to explore targeted treatment avenues, including patient-specific approaches like cancer stem cell-guided chemotherapy and molecular subtype-based strategies (e.g., MGMT-unmethylated GB). The systematic review evaluates key trials, focusing on design, efficacy, and their potential to enhance GB management (summarized in Table 1). Critical findings from these trials highlight advancements in immunotherapy, targeted therapies, and optimized radiotherapy approaches.

**Table 1:** Summary of systematic review for clinical trials evaluating emerging therapies for GB

Study type and duration	Sample size and Age/Sex	Criteria	Intervention	Outcome measures	Key findings	Adverse events	References
Randomized, Double-Blind 7 years	**n** = 447 **Mean Age/Sex:** 58.5, Male/Female	**Inclusion:** Newly diagnosed GBM **Exclusion:** Severe renal impairment	**Arm I** (Temozolomide + Veliparib) and **Arm II** (Temozolomide + Placebo)	Overall Survival (OS), Progression-Free Survival (PFS), Adverse Events, Overall Tumor Response	OS at 83 months (**Arm I**: 24.8 vs. **Arm II**: 28.1 months, P = 0.1462); PFS at 83 months (**Arm I**: 12.1 vs. **Arm II**: 13.2 months, P = 0.3059); OTR (**Arm I**: 15.2%, **Arm II**: 16.6%, P = 0.6827)	All-cause mortality- **Arm I**: 166/212 (78.3%) vs. **Arm II**: 169/217 (77.9%), Neurological Events- Seizures: **Arm I**: 13.2% vs. **Arm II**: 14.8%; Headache: **Arm I**: 51.4% vs. **Arm II**: 45.6%	[92]
Randomized, Open-Label 10 years, 5 months	**n** = 529 **Mean Age/Sex**: 55.4 (Range: 18–75), Male/Female	**Inclusion:** Recurrent GBM **Exclusion:** Severe hepatic impairment	Nivolumab ± Ipilimumab + Standard of Care (RT, TMZ, Surgery) **Control:** Bevacizumab	Overall Survival (OS), Progression-Free Survival (PFS), Adverse Events, Tumor Response	OS at 12 months similar (**Nivo**: 41.8% vs. **Bev**: 42.4%, P = 0.9208); PFS better with **Bevacizumab** (1.51 vs. 3.61 months, HR = 1.88, 95% CI: 1.50–2.35, P < 0.001); ORR (**Nivo**: 7.8%, **Bev**: 23.1%, OR = 0.29, 95% CI: 0.15–0.59)	All-cause mortality- **Nivo**: 180/184 (97.83%) vs. **Bev**: 166/185 (89.73%), Neurological Events- Seizures: **Nivo**: 18.13% vs. **Bev**: 10.30%; Headache: **Nivo**: 31.32% vs. **Bev**: 32.12%	[93]
Randomized, Quadruple-Blind 36 Months	**n** = 78 **Median Age/Sex**: 59 (Range: 18–75), Male/Female	**Inclusion:** Confirmed recurrent GBM, prior chemo failure **Exclusion:** Severe comorbidities, active infection	**Arm I:** Standard Chemotherapy (Physician's Choice) **Arm II:** ChemoID-Guided Chemotherapy	Overall Survival (OS), Progression-Free Survival (PFS), Adverse Events	Significant OS improvement (**Arm I:** 12 vs. **Arm II:** 7.5 months, HR = 0.68, 95% CI: 0.55–0.84, P = 0.007), PFS (**Arm I:** 10.1 vs. **Arm II:** 3.5 months, HR = 0.62, 95% CI: 0.50–0.78, P = 0.002)	All-cause mortality- **Arm I:** 35/35 (100%) vs. **Arm II:** 43/43 (100%), Neurological Events- Seizures: **Arm I:** 2.86% vs. **Arm II:** 2.33%; Neuropathy: **Arm I:** 5.71% vs. **Arm II:** 4.65%	[94]
Randomized, Open-Label 19.3 Months	**n** = 159 **Median Age/Sex**: 60 (Range: 28–79), Male/Female	**Inclusion:** Confirmed GBM with unmethylated MGMT, Karnofsky ≥ 70 **Inclusion:** Severe comorbidities preventing treatment	**Arm I:** Temozolomide + Radiation **Arm II:** Ipilimumab + Nivolumab + Radiation	Progression-Free Survival (PFS), Overall Survival (OS), Adverse Events, QoL Measures	No significant difference in PFS—**Arm I:** 8.5 vs. **Arm II:** 7.7 months, HR = 1.47, 95% CI: 0.98–2.21, P = 0.96)	All-cause mortality- **Arm I:** 41/80 (51.25%) vs. **Arm II:** 37/79 (46.84%), Neurological Events- Seizures: **Arm I:** 20.27% vs. **Arm II:** 39.74%; Headache: **Arm I:** 44.59% vs. **Arm II:** 4.65%	[95]
Randomized, Open-Label 49 Months	**n** = 749 **Median Age/Sex**: 58 (Range: 20–79), Male/Female	**Inclusion:** Confirmed GBM diagnosis, Karnofsky ≥ 70 **Exclusion:** Patients with severe comorbidities preventing treatment	**Arm I:** Marizomib + Temozolomide + Radiation **Arm II:** Temozolomide + Radiation	Overall Survival (OS), Progression-Free Survival (PFS), Mini-Mental State Examination (MMSE), Health-related Quality of Life (HRQoL), Adverse Events	Slight improvement in OS—**Arm I:** 16.13 vs. **Arm II:** 15.08 months, HR = 1.07, 95% CI: 0.98–1.23, P = 0.41), PFS (**Arm I:** 6.34 vs. **Arm II:** 6.11 months, HR = 1.05, 95% CI: 0.89–1.18, P = 0.46)	All-cause mortality- **Arm I:** 55/309 (17.80%) vs. **Arm II:** 57/307 (18.57%), Neurological Events- Seizures: **Arm I:** 11.48% vs. **Arm II:** 12.79%; Headache: **Arm I:** 53.44% vs **Arm II:** 30.98%	[96]
Randomized, Double-Blind 4.5 Years	**n** = 716 **Median Age/Sex**: 58 (Range: 18–75), Male/Female	**Inclusion:** Confirmed GBM diagnosis, Karnofsky ≥ 70, No corticosteroids at baseline **Exclusion:** Severe comorbidities, active autoimmune disease	**Arm I:** Nivolumab + Temozolomide + Radiation **Arm II:** Temozolomide + Radiation + Placebo	Progression-Free Survival (PFS), Overall Survival (OS), 12-Month OS Rate, 24-Month OS Rate, Adverse Events	No significant difference in OS (**Arm I:** 28.91 vs. **Arm II:** vs. 32.07 months, HR = 1.10, 95% CI: 0.91–1.33, P = 0.34), PFS (**Arm I:** 10.64 vs. **Arm II:** 10.32 months, HR = 1.06, 95% CI: 0.90–1.25, P = Not Significant)	All-cause mortality- **Arm I:** 221/355 (62.25%) vs. **Arm II:** 216/354 (61.02%), Neurological Events- Seizures: **Arm I:** 12.96% vs. **Arm II:** 11.30%; Headache: **Arm I:** 1.97% vs. **Arm II:** 1.69%	[97]
Randomized, Open-Label 6 + years	**n** = 560 **Mean Age/Sex**: 57.6, Male/Female	**Inclusion:** Newly diagnosed GBM **Exclusion:** Severe hepatic impairment	**Arm I:** Nivolumab + Radiation Therapy **Arm II:** Temozolomide + Radiation Therapy	Overall Survival (OS), Progression-Free Survival (PFS), Adverse Events, Tumor Response	OS at 3 years (**Arm I:** 13.40 months vs. **Arm II:** 14.88 months, P = 0.0037); HR for OS (1.31, 95% CI: 1.09–1.58); PFS at 6 years (**Arm I:** 6.01 months vs. **Arm II:** 6.21 months, HR = 1.43, 95% CI: 1.19–1.71, P = TBD); 24-month OS rate (**Arm I:** 10.6% vs. **Arm II:** 21.2%)	All-cause mortality- **Arm I:** 269/280 (96.07%) vs. **Arm II:** 253/280 (90.36%), Neurological Events- Seizures: **Arm I:** 13.31% vs. **Arm II:** 10.91%; Headache: **Arm I:** 3.96% vs. **Arm II:** 1.82%	[98]
Randomized, Triple-Blind 15.5 Months	**n** = 691 **Mean Age/Sex**: 58 (Range: 18–75), Male/Female	**Inclusion:** Confirmed GBM with EGFR amplification **Exclusion:** Severe hepatic impairment	**Arm I:** Depatuxizumab Mafodotin + TMZ + Radiation **Arm II:** Placebo + TMZ + Radiation	Overall Survival (OS), Progression-Free Survival (PFS), Adverse Events, Neurocognitive Functioning	No OS improvement (**Arm I:** 18.9 vs. **Arm II:** 18.7 months, HR = 1.02, 95% CI: 0.82–1.26, P = 0.633); PFS (**Arm I:** 8.0 vs. **Arm II:** 6.3 months, HR = 0.84, 95% CI: 0.70–1.01, P = 0.029)	All-cause mortality- **Arm I:** 4/342 (1.17%) vs. **Arm II:** 4/335 (1.19%), Neurological Events- Seizures: **Arm I:** 3.22% vs. **Arm II:** 3.58%; Headache: **Arm I:** 0.29% vs. **Arm II:** 0.0%	[99]
Randomized, Open-Label 24 Months	**n** = 221 **Median Age/Sex**: 60 (Range: 18–75), Male/Female	**Inclusion:** Confirmed recurrent GBM diagnosis, Karnofsky ≥ 70 **Exclusion:** Patients with severe comorbidities preventing treatment	**Arm I:** DSP-7888 + Bevacizumab **Arm II:** Bevacizumab Alone	Overall Survival (OS), Progression-Free Survival (PFS), Objective Response Rate (ORR), Duration of Response (DOR), Adverse Events	Slight improvement in OS (**Arm I:** 10.2 vs. **Arm II:** 9.4 months, HR = 1.12, 95% CI: 0.94–1.29, P = 0.21), PFS (**Arm I:** 5.3 vs. **Arm II:** 3.8 months, HR = 1.18, 95% CI: 1.05–1.34, P = 0.048)	All-cause mortality- **Arm I:** 96/109 (88.07%) vs. **Arm II:** 89/108 (82.41%), Neurological Events- Seizures: **Arm I:** 20.37% vs. **Arm II:** 11.11%; Headache: **Arm I:** 36.11% vs. **Arm II:** 25.56%	[100]
Randomized, Quadruple-Blinded 12 Months	**n** = 35 **Mean Age/Sex**: 55.2 (SD 13.5), Male/Female	**Inclusion:** Recurrent or Progressive GBM **Exclusion:** Standard exclusion criteria for underlying conditions	**Arm I:** Bevacizumab + Chemotherapy + Fractionated Radiosurgery **Arm II:** Standard Chemotherapy	Local Tumor Control, Overall Survival (OS), Progression-Free Survival (PFS)	Survival Rate (Median OS: **Arm I:** 7.2 vs. **Arm II:** 4.8 months; P = 0.11), Progression-Free Survival (Median PFS: **Arm I:** 5.1 vs. **Arm II:** 1.8 months; P = 0.001)	All-cause mortality- **Arm I:** 15/18 (83.33%) vs. **Arm II:** 14/17 (82.35%), Neurological Events- Seizures: **Arm I:** 5.56% vs. **Arm II**: 0.0%; Headache: **Arm I:** 5.56% vs. **Arm II**: 0.0%	[101]
Randomized, Quadruple-Blinded 24 Weeks	**n** = 198 **Median Age/Sex**: 55.1 (Range: 19–84), Male/Female	**Inclusion:** Patients with brain tumor-cognitive impairment **Exclusion:** Severe cognitive deficits prevent the evaluation	**Arm I:** Donepezil Hydrochloride (5 mg, then 10 mg daily) **Arm II:** Placebo	Memory (HVLT-Immediate Recall & Discrimination), QoL, Adverse Events	No significant effect on immediate recall (**Arm I:** 22.5 vs. **Arm II:** 22.2, P = 0.62), but improved discrimination memory (**Arm I:** 10.1 vs. **Arm II:** 9.2, P = 0.007)	All-cause mortality- **Not Reported** Neurological Events- Memory impairment: **Arm I:** 35.42% vs. **Arm II**: 36.96%; Headache: **Arm I:** 43.75% vs. **Arm II**: 43.48%	[102]
Randomized, Open-Label 4.4 Years	**n** = 1173 **Median Age/Sex**: 58 (Range: 21–87), Male/Female	**Inclusion:** Confirmed histologic GBM and MGMT analysis submitted **Exclusion:** Patients with insufficient tissue for MGMT analysis	**Arm I:** Adjuvant TMZ (100 mg/m2 for 5 days of a 28-day cycle) **Arm II:** Dose-Dense TMZ (75 mg/m2 for 21 days—28-day cycle)	Overall Survival (OS), Progression-Free Survival (PFS), MGMT Status, QoL (MDASI-BT, EORTC QLQ-C30)	No significant difference in OS (**Arm I:** 16.6 vs. **Arm II:** 14.9 months; P = 0.); PFS favoring dose-dense TMZ (**Arm I:** 6.7 vs. **Arm II:** 5.5 months); MGMT methylation impacting OS (**Arm I:** 21.4 vs. **Arm II:** 20.2 months)	All-cause mortality- **Not Reported** Neurological Events- Convulsions: **Arm I:** 5.62% vs. **Arm II**: 5.71%; Headache: **Arm I:** 2.20% vs. **Arm II**: 1.67%	[103]
Randomized, Open-Label 7 Years	**n** = 562 **Median Age/Sex**: 73 (Range: 65–90), Male/Female	**Inclusion:** Confirmed histologic GBM and MGMT analysis submitted **Exclusion:** Patients with severe comorbidities impacting treatment eligibility	**Arm I:** Temozolomide (concurrent & adjuvant) **Arm II:** Short Course Radiation	Overall Survival (OS), Progression-Free Survival (PFS), MGMT Methylation, Adverse Events	Temozolomide significantly improves OS (**Arm I:** 9.33 vs. **Arm II:** 7.62 months) and PFS (**Arm I:** 5.29 vs. **Arm II:** 3.94 months), especially in MGMT-methylated patients (**Arm I:** 13.47 vs. **Arm II:** 7.69 months)	All-cause mortality- **Not Reported** Neurological Events- Seizure: **Arm I:** 9.96% vs. **Arm II**: 7.38%; Headache: **Arm I:** 1.85% vs. **Arm II**: 2.58%	[104]
Randomized, Double-Blind 25 Months	**n** = 325 **Median Age/Sex**: 53 (Range: 18–75), Male/Female	**Inclusion:** Confirmed recurrent GBM, no prior Lomustine use **Exclusion:** Severe comorbidities, active infection	**Arm I:** Cediranib (30 mg) **Arm II:** Cediranib (20 mg) + Lomustine **Arm III:** Lomustine + Placebo	Progression-Free Survival (PFS), Overall Survival (OS), Response Rate, 6-Month Progression-Free Rate (APF6), Steroid Reduction	No significant difference in OS (**Arm I:** 8.0 vs. **Arm II:** 9.4 vs. **Arm III:** 9.8 months, HR = NA, P = NA), PFS (**Arm I:** 92 vs. **Arm II:** 125 vs. **Arm III:** 82 days)	All-cause mortality- **Not Reported** Neurological Events- Convulsion: **Arm I:** 8.59% vs. **Arm II**: 2.44% vs. **Arm III:** 3.13%; Headache: **Arm I:** 1.56% vs. **Arm II**: 0.81% vs. **Arm III:** 1.56%	[105]
Randomized, Parallel, Open-Label 12 Months	**n** = 240 **Mean Age/Sex**: 51.2 (SD 11.4), Male/Female	**Inclusion:** Glioblastoma Multiforme, Astrocytoma **Exclusion:** Severe comorbid conditions affecting study participation	**Arm I:** Imatinib Mesylate + Hydroxyurea (HU) **Arm II:** Hydroxyurea Alone	Progression-free survival (PFS), Overall Survival (OS), Adverse Event Rates, Serious Adverse Events (SAEs), Drug-Related Discontinuations	Median PFS: 6 Months (**Arm I:** 5.3 vs. **Arm II:** 6.6), 12 Months (**Arm I:** 2.1 vs. **Arm II:** 2.1); High SAE rates observed in both arms	All-cause mortality- **Not Reported** Neurological Events- Convulsion: **Arm I:** 5.08% vs. **Arm II**: 1.69%; Headache: **Arm I:** 22.03% vs. **Arm II**: 20.34%	[106]
Randomized, Triple-Blinded, Crossover 24 Weeks	**n** = 130 **Mean Age/Sex**: 55.6 (SD 12.2), Male/Female	**Inclusion:** Patients with breakthrough cancer pain using opioids **Exclusion:** Patients with severe cognitive deficits or opioid intolerance	**Arm I:** Fentanyl Sublingual Spray (100–1600 µg) **Arm II:** Placebo	Summed Pain Intensity Differences (SPID), Total Pain Relief (TOTPAR), Global Evaluation, Adverse Events	Fentanyl significantly improves pain relief (SPID30: 640.3 vs. 399.6; TOTPAR60: 176.4 vs. 131.2; Global 60 min score 3.1 vs. 2.2)	All-cause mortality- **Not Reported** Neurological Events- Dizziness: **Arm I:** 7.69% vs. **Arm II**: 2.04%; Headache: **Arm I:** 3.85% vs. **Arm II**: 3.06%	[107]

#### Emerging therapeutic innovations and future directions of GB

The therapeutic approach to managing GB has been evolving with the integration of various therapies to address the invasive nature of the tumor and its elevated recurrence rate. The current therapeutic approaches emphasize combining surgical, radiation intervention, and chemotherapeutic agents to target distinct tumor growth and its dissemination [10]. The emphasis has been placed on enhancing patient outcomes by improving surgical interventions, developing more potent chemotherapeutic agents, and investigating novel treatment approaches.

#### Image-guided surgery

A critical component in GB treatment is surgical resection, which aims to lower tumor progression, diminish neurological deficits, and facilitate adjuvant delivery of drugs. Image-guided surgery plays a pivotal role in improving surgical accuracy and safety with real-time observation of the tumor’s location and surrounding brain tissues [108]. Preoperative surgical planning is essential in determining the feasibility and extent of resection, specifically for tumors present near eloquent brain areas (regions responsible for motor and sensory functioning) [109]. Advanced neuroimaging techniques such as functional MRI (fMRI) and diffusion tensor imaging (DTI)-based tractography are now routinely used to identify and preserve critical functional areas and white matter tracts (e.g., language pathways). This helps minimize the risk of post-operative deficits [110]. Additionally, intraoperative MRI (iMRI), including motor-evoked potentials (MEPs), somatosensory-evoked potentials (SSEPs), and, in selected cases, awake craniotomy with cortical mapping, is employed to provide real-time feedback on neural function during resection, particularly when operating near eloquent cortex [111]. Surgeons might also observe tumor margins with more precision using intraoperative ultrasonography, which utilizes sound waves to provide real-time images [111]. Moreover, intraoperative imaging using 5-aminolevulinic acid (5-ALA) fluorescence improves its ability to discern tumors from healthy tissues by utilizing the fluorescent properties of the dye, which preferentially targets glioma cells [112]. This approach is particularly beneficial in imaging infiltrative tumors, which are challenging to identify through conventional imaging techniques [113]. While modern techniques optimize surgical outcomes and increase the likelihood of achieving maximal safe resection, it’s very crucial to acknowledge their limitations. Complete tumor resection remains rarely achievable, as GB cells infiltrate into surrounding normal brain tissue beyond the visible tumor margin. Furthermore, the availability and implementation of tools such as iMRI and 5-ALA may be constrained by cost and technical requirements. Inadequate surgical resection, especially without image guidance or functional preservation strategies, increases the likelihood of early tumor recurrence through residual infiltrating cells in adjacent healthy tissues [114, 115].

#### Radiotherapy

Radiotherapy is another essential part of GB therapy, specifically in adjuvant circumstances after surgical resection, intending to eradicate residual tumor cells and diminish the probability of recurrence [116]. The significant mechanism behind radiotherapy is stimulating DNA double-strand breaks and the formation of reactive oxygen species (ROS), both of which are very life-threatening to glioma cells. Such forms damage cells, leading to cell death and inhibiting tumor development [117]. To maximize tumor targeting and lower the risk to healthy tissue, a variety of RT treatments have been developed. The foremost RT is the 2D conventional RT that utilizes two or more radiation beams aimed at the tumor [118]. Despite its effectiveness and reasonable pricing, it has very little accuracy compared with modern techniques. 3D conformal techniques are another technique providing an enhancement by modulating the tumor using 3D computer-generated images, enabling the radiation waves to more precisely match the tumor’s form [119]. The precision of this treatment enhances the treatment’s effectiveness by lowering the risk of radiation to neighboring healthy tissues [119]. Emerging radiotherapy approaches for glioblastoma aim to improve precision and therapeutic outcomes while minimizing collateral damage. By modulating the intensity of the radiation beam, Intensity Modulated Radiation Therapy (IMRT) goes a step beyond 3D conformal techniques by modulating the intensity of the radiation beam [120]. It uses computer-controlled radiation beams to shape the radiation dose according to the tumor’s three-dimensional geometry, allowing for higher doses to the tumor while sparing critical areas such as the brainstem and cerebral cortex [121]. Another advanced modality is Proton Beam Therapy, which uses protons instead of X-rays. This technique allows for extremely precise targeting due to the unique physical properties of protons, making it especially beneficial for treating tumors located deep within the brain. Proton therapy minimizes damage to surrounding healthy tissue and may offer better long-term neurocognitive outcomes [122]. In parallel with technological advances, new strategies are being explored that combine radiotherapy with systemic approaches to enhance therapeutic efficacy. These include adoptive immunotherapy, which harnesses the patient’s immune system to attack tumor cells, and targeting molecular pathways such as PI3K, which is frequently altered in glioblastoma [123]. Additionally, combining radiotherapy with chemotherapy or immunotherapy is under investigation to create a synergistic effect and improve patient outcomes [124].

#### Inhibitor-based therapy

Inhibitor-based therapies are novel therapeutic strategies that have revolutionized the management of GB. These therapies target specific pathways that aid in tumor progression, angiogenesis, and the replication of DNA, by providing better accuracy compared to traditional treatments [46]. Angiogenesis enables cancer cells to sustain their rapid proliferation. GB tumors develop an abnormal, immature vascular network characterized by high permeability, irregular branching, and fluctuating lumen diameters, which leads to edema, disrupted blood flow, and increased interstitial pressure. This dysfunctional vasculature not only facilitates tumor cell dissemination but also impairs therapeutic drug delivery, promoting resistance to treatment [125]. VEGF-A, a principal regulator of angiogenesis, plays a crucial role in GB progression. High VEGF expression in hypoxic tumor regions, triggered by hypoxia-inducible factor 1-alpha (HIF-1α), stimulates endothelial cell proliferation and migration, promoting pathological angiogenesis. VEGF exerts its effects via tyrosine kinase receptors VEGFR-1, VEGFR-2, and VEGFR-3, with VEGFR-2 being the most potent mediator of endothelial functions [126]. Given the central role of VEGF in GB angiogenesis, several therapeutic strategies have been developed to inhibit VEGF signaling: Bevacizumab, a monoclonal antibody targeting VEGF-A, reduces tumor vascularity and inflammation. While it improves progression-free survival, its impact on overall survival remains modest. Research suggests that VEGF-A expression levels correlate with response to bevacizumab, and lower doses may offer superior outcomes while minimizing adverse effects. Aflibercept, a recombinant fusion protein designed to neutralize VEGF-A and placental growth factor (PlGF), significantly reduces circulating VEGF levels, impacting tumor response [127]. Biomarkers like MCP3/CCL7 and VEGFR1 + monocytes are associated with improved efficacy, highlighting the potential for personalized treatment approaches [128]. Cediranib is a pan-VEGF receptor tyrosine kinase inhibitor that stabilizes tumor vasculature and improves blood–brain barrier integrity. Cediranib has demonstrated progression-free survival benefits, but it does not significantly enhance overall survival, and resistance mechanisms involving PDGF-C and c-Met remain challenging [129]. Axitinib, a VEGFR tyrosine kinase inhibitor, selectively targets tumor vasculature and GB stem-like cells. It has shown promise in pre-clinical models, reducing tumor vessel density and increasing immune infiltration. However, its clinical efficacy is limited by tumor heterogeneity and blood–brain barrier penetration [130]. Dovitinib, a multi-kinase inhibitor targeting VEGFR, FGFR, and PDGFRβ, theoretically offers broad spectrum angiogenic inhibition. However, clinical trials have shown limited efficacy in recurrent GB, underscoring the need for patient-specific biomarkers and Sunitinib, a multi-targeted kinase inhibitor affecting VEGFR, PDGFR, and other tumor-promoting pathways [131, 132]. While anti-angiogenic therapies have reshaped GB treatment, challenges remain in overcoming resistance mechanisms and improving overall survival. Emerging strategies involve combination therapies, predictive biomarker identification, and novel drug delivery systems such as intranasal administration. Additionally, targeting Rho-associated kinase 2 (ROCK2) may enhance anti-angiogenic treatments, reducing GB cell viability and improving apoptosis rates [133]. Another treatment strategy is PARP inhibitors, e.g., Niraparib, Veliparib, Pamiparib, and Olaparib, which inhibit the enzyme poly (ADP-ribose) polymerase responsible for DNA repair. The inhibition of PARP causes an overabundance of DNA, specifically the breakage of double strands, which tumor cells lack the ability to repair, gradually causing apoptosis of tumor cells [134]. Tyrosine kinase inhibitors, e.g., Erlotinib, Cabozantinib, Imatinib, Gefitinib, Sorafenib, and Dasatinib, target and inhibit enzymes that govern cell signaling pathways like MAPK/ERK involved in cell growth and cell survival of gliomas [130]. Likewise, mTOR inhibitors, for example, Temsirolimus and Everolimus, target mTOR, which is a crucial regulator for tumor cell proliferation and survival [135]. Histone deacetylase inhibitors (HDACis) are another group of drugs that target histone deacetylase and act as an anti-GB activity. Research studies have shown that GB patients have overexpression of HDAC9 levels in the brain. HDAC9 proliferates GB and tumor formation via activation of transcription coactivator having PDZ-binding motif (TAZ), an effector of the Hippo pathway [136]. Certainly, HDACis have also shown significant effects on glioblastoma stem cells; drugs such as valproic acid and romidepsin induce apoptosis of glioma cells and reduce the proliferation rates of GB [137, 138]. The management of GB is improved when it is combined with other treatment approaches. Ultimately, these receptor inhibitor therapies open new avenues in GB treatment, with the potential to address the molecular and genetic basis of tumor growth.

#### Immunotherapy

Immunotherapy is a treatment that employs the body’s immune system to target and eradicate tumor cells systematically. To generate prolonged and more effective responses against GB, various immunotherapies are under investigation [139]. Cemiplimab and Nivolumab belong to the class of checkpoint inhibitors that target the Programmed Death (PD-1) receptor on T cells and inhibit interaction with PDL1, a protein that is responsible for the overexpression of tumor cells evading immune detection [140]. These inhibitors enhance the ability of the immune system to detect and eliminate GB cells by restoring T cells. Another possible approach is vaccine-based therapies, such as Rindopepimut, directed at EGFRvIII mutations frequently identified in GB, which are intended to enhance the immune system to recognize and eradicate tumor cells that exhibit this specific protein [141]. Another vaccine, DCVax-L, facilitates a targeted immune response toward the tumor cells by proliferating dendritic cells to express tumor-specific proteins. Biologically designed genetic viruses, such as VB-111, are implemented in oncolytic viral therapy to specifically infiltrate and eradicate tumor cells [142]. These modified viruses encompass a modified gene that encodes for the chimeric death receptor that activates the body’s immune system and induces the tumor to undergo cell apoptosis. Another promising therapy has been Bispecific T cell engagers and Chimeric antigen receptor-T cell (CAR-T) therapies, in which an individual’s T cells are altered and modified to generate the chimeric antigen receptors that target particular proteins, facilitating tumor cells [143]. Bispecific T cells have shown their great safety and efficacy in pre-clinical models by binding to the CD3ε subunit of T cells and tumor-specific antigens (TAAs), forming a cytotoxic synapse. This synapse leads to the release of perforin and cytotoxic granzyme B, ultimately causing apoptosis of the tumor cells. Genetics and nucleic acids (DNA/RNA)-based vaccines are another intriguing approach in the treatment of GB [144, 145]. TAAs were the intended targets of these vaccines, which involve DNA plasmids and mRNA vaccines. For example, an altered form of the protein associated with tumor angiogenesis, VEGFR-2, is expressed by the VXM01-DNA vaccine [146]. VXM01 demonstrated promising results in recurrent GB patients in a phase I clinical trial, with a few individuals indicating a longer survival time [147]. Furthermore, improved survival was associated with reduced tumor levels of PD-L1 expression, highlighting the potential of incorporating VXM01 with anti-PD-L1 inhibitors might improve the therapeutic effectiveness [148]. Likewise, INO-5401 is another DNA vaccine that integrates antigens that are overexpressed in a variety of malignant tumors, including GB, notably telomerase reverse transcriptase (hTERT), Wilms tumor gene 1 (WT1), and prostate-specific membrane antigen (PSMA). Both CD4 + and CD8 + T cell responses have been clinically studied to be enhanced by these INO-5401 vaccinations, providing an effective immunological target toward GB [146]. The initial findings from ongoing clinical investigations evaluating the safety and efficacy of these specific immunizations are empowering, specifically when matched with immune checkpoint inhibitors [144]. Through employing the body’s immune system, this immunotherapeutic strategy has become a promising novel avenue in addressing GB and its likelihood of a long-term patient outcome.

#### Nano formulation therapy

Nanotechnology is introduced as a revolutionary GB treatment offering novel therapeutic strategies, by increasing effectiveness and lowering systemic toxicities [149]. Nanotech formulations such as liposomes exhibit distinct potential in this area, as they are lipid-based carriers for drugs to be delivered directly to the target cell. Liposomes also increase the therapeutic bioavailability of the drugs, minimizing their errors of binding at off-target receptors [150]. Liposomal irinotecan, a chemotherapeutic drug encapsulated in nanoformulation—liposomes, is a prominent instance of this therapy and is given using convection-enhanced delivery (CED). This technique improved the drug’s permeability by infusing it directly into the brain, crossing the BBB, and enhancing treatment effectiveness by reducing the probable causes of systemic toxicity. To improve non-invasive brain delivery and better tumor targeting, a unique nanocapsule has been designed, namely CRISPR-Cas9, with promising effects in targeting gliomas and demonstrating a secure drug delivery system [151]. There, another research study was carried out using exosome-based nanocarriers for their treatment drug doxorubicin (Pep2-Exos-Dox) targeting GB [152]. This exosome was synthesized from an oligonucleotide of BV2 microglial cells, this method improved its BBB penetration and better drug release in circulation [153]. Despite the significant advancements in nanotechnology-based therapies for GB, several physicochemical limitations continue to challenge their clinical translation. One major concern is off-target accumulation, where nanoparticles may localize in non-tumor tissues, potentially leading to systemic toxicity [154]. Additionally, effective penetration of the BBB remains inconsistent and often depends heavily on the nanoparticle’s size, surface charge, and functionalization. Some formulations may also induce ROS, triggering oxidative stress and cellular damage [155]. Furthermore, immune recognition of nanoparticle-based systems can provoke inflammatory responses, complicating repeated dosing strategies. The long-term safety profile and potential for tumor cells to develop resistance to certain nanoparticle carriers are also under investigation [156]. Addressing these challenges is essential for optimizing nanoparticle design and ensuring their safety and efficacy in clinical settings. Nano techniques have enormous potential to improve treatment outcomes for GB by leveraging the unique characteristics and nature of improved drug delivery mechanisms, enabling a more targeted and efficient therapeutic strategy to combat GB [113].

## Conclusion

GB remains one of the most aggressive and complex brain disorders, necessitating continuous advancements in molecular profiling, signaling pathways, and pathophysiology to develop novel therapeutic strategies. Early diagnosis plays a crucial role in improving survival rates, emphasizing the need for enhanced diagnostic approaches and histopathological precision. Current treatment modalities, including surgical interventions, immunotherapies, radiotherapies, and chemotherapies, have provided some relief, but emerging techniques such as nanotechnology, monoclonal antibodies, and vaccine development hold promise for improving patient outcomes and quality of life. Despite significant strides in GB research, challenges persist, particularly in overcoming tumor resistance, blood–brain barrier limitations, and treatment heterogeneity. The integration of combinatorial therapies and clinical research is essential to refine treatment protocols and develop more effective, personalized approaches. As research continues to evolve, the future of GB therapy lies in precision medicine, advanced drug delivery systems, and multi-modal strategies that address the disease’s complexity. While progress has been made, the battle against GB is far from over, requiring sustained innovation and collaboration to improve long-term survival and patient care.

## Data Availability

No datasets were generated or analyzed during the current study.
